# Prehospital Advanced Diagnostics and Treatment of Acute Stroke: Protocol for a Controlled Intervention Study

**DOI:** 10.2196/resprot.8110

**Published:** 2018-02-28

**Authors:** Kristi G Bache, Maren Ranhoff Hov, Karianne Larsen, Volker Moræus Solyga, Christian G Lund

**Affiliations:** ^1^ Research and Development The Norwegian Air Ambulance Foundation Drøbak Norway; ^2^ Institute of Basic Medical Sciences Faculty of Medicine University of Oslo Oslo Norway; ^3^ Department of Neurology Oslo University Hospital Oslo Norway; ^4^ Department of Neurology Østfold Hospital Kalnes Norway

**Keywords:** biomarkers, blood analysis, stroke scales, cerebral CT examinations, rtPa, air ambulance model, MSU

## Abstract

**Background:**

Acute ischemic stroke (AIS) is a medical emergency. The outcome is closely linked to the time elapsing from symptom onset to treatment, and seemingly small delays can mean the difference between full recovery and physical and cognitive dysfunction. Recanalization to allow blood to reenter the affected area is most efficient immediately after symptoms occur, and intravenous thrombolysis must be initiated no later than 4.5 hours after the symptom onset. A liable diagnosis is mandatory to administer the appropriate treatment. Prehospital diagnosis and, in cases where contraindications are ruled out, prehospital initiation of intravenous thrombolysis have been shown to significantly decrease the time from alarm to the treatment.

**Objective:**

The objective of this paper is to investigate the effectiveness of prehospital thrombolysis as measured by (1) time spent from symptom onset to treatment and (2) the number of patients treated within 4.5 hours. In addition, we want to conduct explorative studies. These will include (1) the use of biomarkers for diagnostic and prognostic use where we will collect blood samples from various time points, including the hyperacute phase and (2) the study of magnetic resonance imaging (MRI) images at day 1 to determine the infarct volume and if the time to thrombolysis has an influence on this.

**Methods:**

This is a prospective controlled intervention study. The intervention will involve a computed tomography (CT) and thrombolysis in a physician-manned ambulance called a mobile stroke unit (MSU). The control will be the conventional pathway where the patient is transported to the hospital for CT, and thrombolysis as per current procedure.

**Results:**

Patient inclusion has started and a total of 37 patients are enrolled (control and intervention combined). The estimated time to completed inclusion is 36 months, starting from May 2017. The results of this study will be analyzed and published at the end of the trial.

**Conclusions:**

This trial aims to document the feasibility of saving time for all stroke patients by providing prehospital diagnostics and treatment, as well as transport to appropriate level of care, in a safe environment provided by anesthesiologists trained in prehospital critical care.

**Trial Registration:**

ClinicalTrials.gov NCT03158259; https://clinicaltrials.gov/show/NCT03158259 (Archived by WebCite at http://www.webcitation.org/6wxNEUMUD)

## Introduction

### Background

Stroke is the third leading cause of death in most Western countries. Two-thirds of stroke survivors struggle with moderate to severe disability [1]. Stroke affects approximately 15 million people worldwide each year. Of these, 5 million die and at least another 5 million are left permanently disabled [2]. In Norway, around 15,000 people suffer from stroke each year, and in the next 20 years, the number of stroke victims will increase substantially [3].

Up to 90% of all strokes are ischemic (cerebral infarction), mostly, due to an acute thromboembolic obstruction of a cerebral artery. The remaining 10% are cerebral hemorrhages. In most ischemic strokes (ISs), a small or a large part of the brain (the infarct core volume) will undergo necrosis within few minutes due to hypoxia. However, because of cerebral collateral flow, a significant brain volume (the penumbra volume) may survive for some hours, but this relies on the rapid restoration of the blood flow [4]. Therefore, an AIS is a medical emergency, and successful treatment relies on early recanalization of the obstructed artery. Thrombolytic therapy (recombinant tissue-type plasminogen activator, rtPA) is approved for use within 4.5 hours after symptom onset [5,6], but the efficacy decreases rapidly with time. This is illustrated by the increase in the number needed to treat (NNT) from 4 within 90 min to more than 19 after 4.5 hours [7] *.* Due to delays and late arrival, only 15% to 40% of all the stroke patients reach the hospital within the designated 4.5 hours, and only about 5% of these patients receive thrombolytic therapy. Even among this 5%, the majority of these patients are treated in the less-efficient end of the approved time window [8].

Differentiation of a cerebral infarction from a cerebral hemorrhage relies on the computed tomography (CT) or the magnetic resonance imaging (MRI) of the brain. This is crucial because thrombolytic treatment of a patient with acute cerebral hemorrhage may be fatal. Therefore, intravenous (IV) thrombolysis is only administered in a hospital at present. The consequence is a multifactorial delay. As a result, very few patients are treated within the most effective period for this drug, which is up to 90 min after the symptom onset. The only way to avoid this detrimental time delay seems evident—establish the diagnosis and the treatment of an AIS outside the hospital and in time as close to symptom onset as possible.

An increasing amount of evidence shows that minimizing prehospital time delay improves the thrombolytic rates in an AIS [9,10], and to take it a step further, in 2012 Fassbender et al demonstrated that prehospital stroke diagnosis is accurate and feasible. Using a mobile stroke unit (MSU), equipped with a stroke neurologist, a CT scanner, and a point-of-care biochemical laboratory, they showed that the time from symptom onset to a diagnostic therapeutic decision for thrombolysis was reduced from 76 min to 35 min. In 95% of the cases, the CT scanner in the MSU provided high-quality brain scans, which enabled them to rapidly and accurately differentiate between cerebral infraction and cerebral hemorrhage on site [8]. Due to the potentially great socioeconomical gain of early treatment of stroke patients [11], other initiatives to investigate and implement a neurologist-staffed MSU have been made [12-14]. They all have in common that they significantly reduce time to diagnosis and treatment. Thus, the question arises if a full hospital staff, including a neurologist, who normally do not operate in the prehospital room, is mandatory for this system to work. To investigate this, we have staffed an MSU with a specially trained anesthesiologist, a specially trained nurse, and a paramedic to mimic the Norwegian helicopter emergency medical service (HEMS) (REK id 2013/2298). On the basis of this, Hov et al published a study on the agreement between anesthesiologists and neuroradiologist in finding radiological contraindications of thrombolytic therapy in cerebral CT scans of an acute stroke [15]. This study showed a 92% interrater agreement between the 2 groups, and it demonstrates that a prehospital diagnosis of stroke patients is feasible on a more general basis and within the Norwegian physician manned emergency service.

The National Institutes of Health Stroke Scale (NIHSS) is a reliable and a much-used tool for clinical recognition and severity estimation of stroke symptoms. The reliability of this scale has been established by several clinical trials performed by trained neurologists. In 1999, Dewey et al proved that the overall agreement in NIHSS scoring between trained nurses and trained neurologists was no different from the agreement among neurologists. The study suggested that trained nurses could administer the NIHSS with reliability similar to stroke-trained neurologists [16]. Thus, the use of NIHSS is accessible in the prehospital system, where specially trained paramedics, nurses, and anesthesiologists can evaluate the patients using NIHSS. This will help with triage and categorization of patients for this study.

The most commonly used brain-imaging method for acute stroke is CT; however, the detection of an ischemic volume is not sensitive in the acute phase. The size of the infarct core can be approximated by the extent of diffusion-weighted imaging (DWI) signal changes [17]. The MRI volumetric in predicting infarct volume in postischemic stroke patients is promising. Recent studies have shown that a large initial DWI lesion volume was an independent predictor of poor outcome in patients managed with intravenous thrombolysis [18], and that lesion volume may decrease more than 30% 2 hours after tissue plasminogen activator (tPA) as an early marker of long-term clinical benefit of thrombolytic therapy [19]. Our aim is to test whether early thrombolysis (< 90 min) may cause lesser infarct volume on DWI MR compared with late thrombolysis (>120 min) and to test DWI MR infarct volume as a predictor of outcome in patients treated with prehospital thrombolysis.

Biomarkers are molecules released by specific organs or types of cells. On the basis of how injuries occur at a cellular level, biochemical markers in the blood after an acute stroke may offer a possibility to gain prognostic, diagnostic, and even therapeutic information. Inflammation in the form of proinflammatory cytokine production, microglia activation, and recruitment of other immune cells after a stroke plays an important role in the pathogenesis [20]. Additionally, as the thrombolytic treatment is extremely sensitive to time and can prevent damage to the brain tissue, pharmacological interventions must be investigated. The extent of damage after a stroke is closely linked to the time elapsed from symptom onset to treatment, and the influence of this time span might be reflected in the inflammatory response measured by the circulating cytokines in the blood. By studying this, we might gain insight into the outcome and the prognostic values after a stroke.

On the diagnostic side, biomarkers have the potential to distinguish an intracerebral hemorrhage (ICH) from an AIS and a stroke mimic [21]. Concentrations and ratios of such markers may be used as a diagnostic tool, and further investigations are needed to fully utilize these possibilities. It has been shown that as much as a 1-month of additional disability-free life can be obtained by every 15-min decrease in treatment delay [22]. This emphasizes the importance of early diagnosis and treatment onset. The ability to diagnose a stroke already in the prehospital phase makes treatment possible at a much earlier time point than today and renders the search for such methods an important and relevant topic.

The use of biomarkers, as a diagnostic tool, to determine whether a person has suffered from an ICH or an AIS remains a challenge but is in progress. Proteins representative of early pathways involved in the pathophysiology of cerebral ischemia have failed to show sufficient diagnostic accuracy [23,24]. Attempts to identify biomarkers specific to ICH have been made, with a focus on the cell-type-specific proteins that are released upon brain damage. The glial fibrillary acidic protein (GFAP) is a structural protein specifically expressed in astrocytes [25], a type of glial cell that performs a variety of signaling and nonsignaling functions in the brain. Upon cellular disintegration, caused by ICH, the GFAP is rapidly released and can be detected in the plasma. Under the physiological conditions, the GFAP is not secreted from the cells; therefore, it is not detected in the plasma [26]. The plasmatic retinol-binding protein 4 (RBP4) was picked up on a big screen that aimed to identify new biomarkers to differentiate the stroke subtypes. In combination with the GFAP, it shows great potential for distinguishing between an AIS and an ICH [27]. By comparing the results from the neuroimaging and the biomarker measurements, we can elucidate the information that the different concentrations and ratios of the mentioned biomarkers in the blood provide. In turn, we hope this will enable us to establish a biochemical means to define the type and the magnitude of the stroke, which is mandatory before the initiation of a treatment.

### Objective

The objective of this study is to investigate the effectiveness of a prehospital diagnosis and, when appropriate, of intravenous thrombolytic treatment of an AIS. At the same time, we will take the opportunity to do an explorative study with the aim to further improve the intervention using biomarkers and volumetric outcome measures measured using MRI images.

The intervention study aims to:

- determine the time from symptom onset to thrombolytic treatment in the MSU compared with the conventional model;
- determine the number of patients receiving thrombolytic treatment within the 4.5-hour window in the MSU compared with the conventional model; and
- determine if thrombolytic treatment in the MSU, when adjusted for time, offers better Modified Rankin Scale (mRS) and Barthel outcome compared with treatment in the conventional model.

The explorative study aims to:

- determine if final AIS infarction volume, estimated by an MRI, is independently correlated with time from symptom onset to thrombolytic treatment;
- define cutoff values for GFAP and RBP4 and explore whether they can distinguish an ICH from an AIS when combined with sufficient specificity and sensitivity; and determine the influence of time to treatment on proinflammatory markers after stroke

### Hypothesis

#### Intervention Study

1. The Treat-NASPP MSU model is feasible and reduces the onset to treatment time (>15 min).
2. The number of patients treated with thrombolysis within 4.5 hours of symptom onset are significantly increased in the Treat-NASPP MSU model.
3. The treatment in the Treat-NASPP MSU model, when adjusted for time, does not result in increased day 90 mRS and Barthel as compared with the conventional model.
4. Prehospital thrombolytic treatment of stroke does not increase the risk of secondary cerebral bleeding as compared with inhospital thrombolytic treatment of stroke (cerebral bleeding worsening within 36 hours <4%, Norsk hjerneslagregister)

#### Explorative Study

1. The final infarct volume, estimated by an MRI, is significantly reduced when the thrombolytic treatment is initiated in the MSU.
2. Biomarkers are a valid tool in the hyperacute phase of cerebral illness to exclude contraindication to thrombolysis.
3. Reduced onset to treatment time results in lower levels of selected proinflammatory molecules.

## Methods

Treat-NASPP is a prospective controlled intervention study. The main aim of this study is to prospectively compare patients with an AIS, who are diagnosed and treated prehospitally in the MSU (intervention), with those who receive conventional pre- and inhospital diagnostics and treatment (control). At the same time, we will perform an explorative study with the aim to further improve the diagnostic (the biomarker study) and the outcome measures (the biomarker study and the MRI infarction volume study).

The MSU will be available on call on weekdays from 8 AM to 8 PM, 2 weeks on and 2 weeks off. The MSU will not be on call during holidays and vacations. When on a call, the MSU will be staffed with an anesthesiologist, a paramedic-nurse, and a paramedic. During the weeks that the MSU is not operating, data collection will take place from the conventional ambulance on weekdays from 8 AM to 8 PM. Only ambulances that are staffed with a paramedic-nurse and a paramedic who work as staff on the MSU will participate in the study. All emergency calls to the central emergency medical services (EMS) dispatch center (AMK 113) from the catchment region of Østfold County will be screened for stroke symptoms by the EMS dispatcher, as per normal procedures—they will use the Functional Assessment Staging Test scale (one or more of the following neurological deficits: paralysis of arm or leg, facial paralysis, aphasia, or dysarthria) and in accordance to the inclusion criteria (listed below), the MSU will be dispatched when the inclusion criteria are fulfilled on weeks when the MSU is on call. The same inclusion criteria will be applied for the ordinary ambulances on weeks when the MSU is not on call. The intervention (prehospital CT and thrombolytic treatment) can only be administered in the MSU. The control (inhospital CT and thrombolytic treatment) can only be administered in the hospital. The EMS dispatcher will notify the EMS service (MSU anesthesiologist or conventional ambulance staff) with clinical information and history, if available. The procedure is outlined below.

Inhospital NIHSS, Barthel, and mRS in the hyper-acute phase will be conducted by the neurologist on call. Follow-up tests will be conducted by an independent, inhospital neurologist who is not invested in the study.

### MSU Procedure

On site, the anesthesiologist will take the actual medical history and conduct a rapid screening using the ABCDEs of trauma care. If the patient is stable and further investigations can proceed, including NIHSS scoring, the patient will get 2 venous lines, and blood samples will be collected. Blood samples for biomarkers (see details below) will be stored and delivered to the laboratory at the hospital for further analyses and storage in a biobank (related to REK 2014/1161). The patient will travel in the stroke ambulance, where the CT scan will be performed and blood tests will be run in the point-of-care laboratory (POC). After completing the CT scan examination, the anesthesiologist will immediately get in contact with the on-call neurologist (stroke team) at the Østfold Hospital. The anesthesiologist will provide the stroke team with the clinical history, the POC blood tests, the NIHSS score, the time of symptom onset, and any known clinical contraindication of thrombolysis. The stroke team and the on-call radiologist will interpret the CT scan by teleradiology, and a treatment decision will be made. If there is an indication of thrombolytic treatment, the stroke ambulance nurse will prepare and initiate intravenous rtPa (Actilyse). The anesthesiologist will fill the prehospital study data in an electronic study form.

### Conventional Ambulance Management

Conventional ambulance data collection will only take place when the MSU is not on duty. The conventional ambulance will be staffed with the same personnels (except the anesthesiologist) as the MSU. After the paramedic or nurse-paramedic has taken the patient’s actual medical history, performed a physical examination, including NIHSS, established a venous line, and given the emergency treatment needed, the patient will be transported to the Østfold Hospital. The paramedic will contact the EMS dispatcher and inform about the patient’s inclusion in the study, and the EMS dispatcher will contact the hospital stroke team. The paramedic will withdraw a blood sample for the biomarker study, and an additional blood sample will be taken at the hospital for standard analysis.

### Therapeutic Decision

In both, the conventional and the MSU pathway, a cerebral CT scan will be conducted as soon as possible after the symptom onset. Images from both the pathways will be registered in the hospital PACS system, and they will be interpreted by the on-call stroke team (the neurologist and the radiologist). In the MSU pathway, the anesthesiologist will decide which patients are eligible for CT scan. However, in the conventional pathway, the on-call neurologist in the emergency department will make this decision. Prehospital clinical assessment will include the actual medical history, a stroke scale score, and an ABCDE evaluation. The clinical information and scores will be completed in designated study forms, and in the specialized prehospital patient record system—AMIS. Both the paramedic/nurse in the conventional pathway and the anesthesiologist in the MSU will do a stroke scale score, and the scores will be analyzed for research purposes.

If a thrombolytic-treated patient shows signs of clinical detoriation, expressed as an increase of 4 or more points on the NIHSS scale, an intracranial hemorrhage would be suspected and the thrombolytic infusion would be stopped immediately. A new cerebral CT scan should be conducted in the MSU if the driving distance to the hospital exceeds 20 min, and these CT findings should be transferred and reported to the hospital immediately. On the basis of the CT image, after the identification of the location and the distribution of damage, the treatment will either be initiated on site, in accordance with inhospital procedures, or the patient will be transported directly to the location for neurosurgery.

### Prehospital Use of Stroke Scales

Stroke scales will be conducted in the MSU and the regular ambulance. All participants (anesthesiologists, paramedics, and nurses) will attend a 2-day course in stroke clinics, stroke treatment, and the use of stroke scales. An online certification in NIHSS will be mandatory for participation.

### Magnetic Resonance Volumetric

An MRI will be completed at day 1 in all patients treated with thrombolysis—prehospital or inhospital. According to the standard MRI protocol at Østfold Hospital, the *final* infarct volume will be estimated using T1-volume, FLAIR, T2, diffusion, and SWI series.

### Biomarker

All serum and plasma samples will be stored in a biobank (related to REK 2014/2261). Venous blood for measurements of biomarkers will be drawn from all the enrolled patients at the earliest time points after symptom onset (ie, after arrival of the paramedics/anesthesiologists at the scene) and at different time points (up to weeks after being admitted in the hospital). The total volume of blood drawn will not exceed 100 mL on any day. All samples will be drawn by personnels certified by the Østfold Hospital. One standard serum tube, 1 EDTA plasma tube, and 1 citrate serum tube will be used for blood collection. Blood samples will be centrifuged within 2 hours of blood collection using a standard centrifuge (10 min at 1500 g-2000 g). Serum/plasma will be transferred immediately in aliquots to Eppendorf tubes (each containing 0.5 mL) and stored at −80°C. The Eppendorf tubes will need to be labeled appropriately mentioning the patient number and the number of tubes collected per patient. Periodically (depending on the number of patients and tubes collected), biomarkers will be analyzed in Professor Sandip Kanses’ lab, IMB, UiO, or shipped on dry ice to Professor Christian Förch, Department of Neurology, Schleusenweg 2-16, 60528 Frankfurt am Main, Germany. Acute CT scans will be taken at the same time as blood samples and will be collected prehospitally (this applies for the MSU. Patients brought in by a conventional ambulance will have their CT scans taken after arrival at the hospital). We will compare biomarker levels (index test) with CT findings (reference standard) and optimize cutoff points by using Receiver Operating Characteristics (ROC) analysis. Sensitivity and specificity will be calculated based on cross tabulations. In the wake of a stroke, the broad range of time points will be needed to monitor the rise and fall of biomarker concentrations, inflammatory response, and diagnostic and prognostic windows.

### Inclusion

- All patients suspected of having a stroke that are checked by the emergency services within 4 hours of symptom onset.
- Patients experiencing stroke symptoms—sudden weakness of leg or arm, especially on one side; facial asymmetry; sudden trouble walking; and speech disturbance (Norwegian Index of Medical Emergencies 27.03-27.06).

### Exclusion

- Under 18 years of age
- Pregnancy
- Female <50 years and uncertain of pregnancy
- Uncertainty regarding symptom onset time

### Study Variables

- Age
- Gender
- Prehospital NIHSS
- NIHSS inhospital (day 0, 2 hours after rtPA, day 1, and day 7)
- mRS (at discharge but no later than day 7, day 30, and day 90) and Barthel score (at discharge but no later than day 7, day 30, and day 90)
- Hyperacute CT diagnosis
- CT-angiography (CTA) findings
- MR volumetric at day 1 and day 90
- Time span from symptom onset to MSU/conventional ambulance admission
- Onset to treatment (symptom onset to thrombolysis)
- Onset to thrombectomy/neurosurgery time

Hemorrhagic transformation categorized as:

- Hemorrhagic infarction 1 (HI1) (small petechiae along the margins of the infarct)
- Hemorrhagic infarction 2 (HI2) (confluent petechiae within the infarcted area but no space-occupying effect)
- Parenchymal hemorrhage (PH1) (blood clots in 30% or less of the infarcted area with some slight space-occupying effect)
- Parenchymal hemorrhage (PH2) (blood clots in more than 30% of the infarcted area with substantial space-occupying effect)
- Remote parenchymal hemorrhage (rPH) (bleeding outside the infarcted area)

- Biomarker concentrations at Time 1 (in MSU and conventional ambulance) and 2 (inhospital)
- History of known comorbidity
- History of anticoagulation

### Data Monitoring, Harms, and Auditing

An independent safety committee consisting of 2 experienced stroke neurologists will review all safety data after 10, 20, 50, 100, 150, and 200 patients are treated with thrombolysis. The committee will stop the study if they find evidence for an unacceptable increase of symptomatic cerebral bleedings (more than 4%) or deaths. Symptomatic cerebral hemorrhage is assessed as a local or a remote parenchymal hemorrhage combined with a neurological deterioration of 4 points or more on the NIHSS from baseline or from the lowest NIHSS value between baseline and 24 hours, or a significant clinical worsening linked to the bleeding or a bleeding leading to death.

The overall rate of cerebral bleeding complications and the mortality rate at 7 days will be compared with the data from the Norwegian stroke register.

The main safety issues in the acute phase of stroke are linked to respiratory failure, cardiac arrhythmias, and cerebral bleeding secondary to thrombolytic treatment.

The anesthesiologists working in the MSU are highly qualified to take care of acute respiratory and cardiac failure. The MSU is technically equipped as an air ambulance helicopter.

The main aim of our study is to provide early thrombolytic treatment to patients with cerebral infarction. The most serious complication of thrombolytic treatment is cerebral bleeding, which may be fatal. Up to 10% of all patients treated with thrombolysis will have a cerebral bleeding confirmed by CT, whereas only 2% to 4% of all patients will die or have a worsened outcome due to bleeding (called symptomatic bleeding). Cerebral bleeding following thrombolytic therapy will show up during the first few days, sometimes even in the very acute phase. Studies with thrombolytic therapy in MSU models have, however, not shown an increased risk of cerebral bleeding [12].

Inhospital cerebral bleeding will be diagnosed and treated according to the standard routines. Patients will be monitored both in the MSU and in the stroke unit with NIHSS scoring at close intervals for 24 to 36 hours. In the hospital, as a routine, an MRI cerebral scan will be performed at approximately 24 hours after symptom onset.

### Statistical Analysis

The Treat-NASPP is designed in accordance with the Standards of Reporting of Diagnostic Accuracy initiative guidelines [28]. For the prospective controlled intervention study, our primary outcome will be (1) onset to treatment time and (2) number of patients treated within 4.5 hours. Our secondary outcome will be mRS and Barthel at day 90 adjusted for onset to treatment time.

For the primary outcomes, we will use the Mann–Whitney *U* test. For power calculation, we want to compare two continuous variables in two groups or compare two means. 2-sample, 2-sided equality. If we estimate the time saved by MSU-treatment (intervention) as compared with the conventional pathway (control) and found the following outcomes:

- Mean group 1: 210 min [29]
- Mean group 2: 180 min (we estimate that 30 min are saved in the MSU)
- SD 70 min
- Sampling ratio: 1

This gives us a number (n) of 86 patients with thrombolytic treatment in each group. As we observed a mean reduction of time from onset of symptoms to diagnostics in the referred to NASPP study (unpublished data, REK 2013/2298) of 100 min, we consider this to be achievable.

As we expect concentrations of biomarkers to have a skewed distribution [21,30], we will use the Mann–Whitney *U* test for comparing concentrations in the patients with an AIS and an ICH. The ROC-curve analysis will be used to calculate the diagnostic accuracy of the biomarkers in distinguishing between an AIS and an ICH.

Sample size calculation for logistic regression is a complex problem, but based on the work of Peduzzi et al [31], the following guideline for a minimum number of cases to include in the study can be suggested:let p be the smallest of the proportions of negative or positive cases in the population and k the number of covariates (the number of independent variables), then the minimum number of cases to include is: N=10 k/p. A statistician (JR) will be consulted for correct data analysis.

### Data Storage

Clinical data will be registered and stored at the Østfold Hospital, Kalnes. Study data will be retrospectively collected and registered using the European Cerebrovascular Research Infrastructure (ECRI) database located at the Oslo University Hospital [32]. ECRI is a platform for European stroke research centers, and it provides the essential infrastructure for international cooperation with shared databases, secure network access, and advanced consent handling. The ECRI was established to facilitate high-quality medical research with possibilities of international cooperation.

### Consent

The Treat-NASPP Study will be closely linked to the NASPP and Biomarker study (REK 2013/2298 and 2014/1161). This biobank will be a continuation of the specific biobank approved by REK related to project 2014/1161. It is stated that the consent may be collected retrospectively when needed (REK document-id: 436501). Patients (or next of kin) included in the study will be informed about the study and an oral consent in the prehospital acute phase will be obtained when possible. In the stroke unit, before discharge, a specifically assigned neurologist will be in charge of collecting written consent from all patients (or next of kin), who are part of this study. This will be closely monitered by the principle investigator and the responsible PhD candidate.

## Results

Patient inclusion has started and a total of 37 patients are enrolled (control and intervention combined). The estimated time to completed inclusion is 36 months, starting from May 2017. The results of this study will be analyzed and published at the end of the trial.

## Discussion

Patients suffering from an AIS can have a complete functional and cognitive recovery or suffer from severe disability and death. The outcome prognosis is strongly associated with successful reperfusion treatment [33]. Diversion of suspected traumatic brain injury patients to trauma centers or patients with identified intracranial hemorrhage may improve outcomes by expediting access to specialist neurosurgical care [34]. Prehospital recognition of symptoms and/or diagnostic findings that resemble the need of endovascular thrombectomy or care in neurosurgical department may be transported directly to the regional hospital by the MSU, or by the conventional ambulance, or the HEMS by-passing the local hospital. The Treat-NASPP Study is the first to introduce advanced prehospital diagnostics and treatment of AIS in a well-established prehospital setting run by anesthesiologists.

This study might result in a method that can be used to diagnose a stroke and initiate treatment prehospitally, which might have a significant clinical impact on the patient outcome.

## Abbreviations

AIS: acute ischemic stroke
CT: computed tomography
ECRI: European Cerebrovascular Research Infrastructure
EMS: emergency medical services
GFAP: glial fibrillary acidic protein
HEMS: helicopter emergency medical service
ICH: intracerebral hemorrhage
IS: ischemic stroke
MRI: magnetic resonance imaging
mRS: Modified Rankin Scale
MSU: mobile stroke unit
RBP4: retinol-binding protein 4
ROC: Receiver Operating Characteristics
rtPA: recombinant tissue-type plasminogen activator
